# Identifying patient‐centred recommendations for improving patient safety in General Practices in England: a qualitative content analysis of free‐text responses using the Patient Reported Experiences and Outcomes of Safety in Primary Care (PREOS‐PC) questionnaire

**DOI:** 10.1111/hex.12537

**Published:** 2017-02-28

**Authors:** Ignacio Ricci‐Cabello, Lorena Saletti‐Cuesta, Sarah P. Slight, Jose M. Valderas

**Affiliations:** ^1^ Nuffield Department of Primary Care Health Sciences University of Oxford Oxford UK; ^2^ National Scientific and Technical Research Council (CONICET) Culture and Society Research and Study Centre (CIECS‐CONICET‐UNC) Córdoba Argentina; ^3^ Division of Pharmacy School of Medicine, Pharmacy and Health Durham University Stockton on Tees UK; ^4^ Department of Pharmacy Newcastle upon Tyne Hospitals NHS Foundation Trust Newcastle UK; ^5^ Division of General Internal Medicine Centre for Patient Safety Research and Practice Brigham and Women's Hospital Harvard Medical School Boston MA USA; ^6^ Health Services and Policy Research Group Patient Centred Care University of Exeter Collaboration for Academic Primary Care (APEx) University of Exeter Medical School University of Exeter Exeter UK

**Keywords:** health services research, patient safety, primary health care, qualitative research

## Abstract

**Background:**

There is a growing interest in identifying strategies to achieve safer primary health‐care provision. However, most of the research conducted so far in this area relies on information supplied by health‐care providers, and limited attention has been paid to patients’ perspectives.

**Objective:**

To explore patients’ experiences and perceptions of patient safety in English general practices with the aim of eliciting patient‐centred recommendations for improving patient safety.

**Methods:**

The Patient Reported Experiences and Outcomes of Safety in Primary Care questionnaire was sent to a random sample of 6736 primary care users registered in 45 English practices. We conducted a qualitative content analysis of responses to seven open‐ended items addressing patients’ experiences of safety problems, lessons learnt as a result of such experiences and recommendations for safer health care.

**Results:**

A total of 1244 (18.4%) participants returned completed questionnaires. Of those, 678 (54.5%) responded to at least one open‐ended question. Two main themes emerged as follows: (i) experiences of safety problems and (ii) good practices and recommendations to improve patient safety in primary care. Most frequent experiences of safety problems were related to appointments, coordination between providers, tests, medication and diagnosis. Patients’ responses to these problems included increased patient activation (eg speaking up about concerns with their health care) and avoidance of unnecessary health care. Recommendations for safer health care included improvements in patient‐centred communication, continuity of care, timely appointments, technical quality of care, active monitoring, teamwork, health records and practice environment.

**Conclusion:**

This study identified a number of patient‐centred recommendations for improving patient safety in English general practices.

## 1 INTRODUCTION

Patient safety, defined by the World Health Organization as “the prevention of errors and adverse effects to patients associated with health care,”^1^ is a clear priority for most health‐care systems.^2^ Research on patient safety has been largely centred around hospitals,^3^ and primary care has been perceived as a low technology environment where safety would not be a problem. However, in England, 90 per cent of contacts with the National Health Service take place in primary care, and more than 750 000 patients consult their GP each day.^4^ A recent systematic review including studies from 21 different countries^5^ estimated that 2‐3 patient safety incidents occur per 100 primary care consultations, and 4% of them result in severe harm (long‐term physical or psychological problems or death). In the UK, this would translate to between 15 000 and 22 500 safety incidents per day, resulting in between 600 and 900 patients being severely harmed each day. Between 45% and 76% of these incidents could be prevented.^6^

Most of the research conducted so far in the area of primary care patient safety is based on information supplied by health‐care providers,^7^ and limited attention has been paid to patients’ perspectives.^8^, ^9^, ^10^ Patients are the common element across the various settings, organizations and health professionals usually involved in their health care, and therefore, they are ideally suited to reflect on the health care they receive. As highlighted by World Health Organization in a recent report,^11^ tapping into such a rich resource could contribute significantly to improving safety in primary care.

A number of recent qualitative studies have examined patients’ perceptions of different aspects of patient safety in primary care, including the ways in which patients make sense of “safety” in the context of primary medical care,^12^ their perceptions of errors in long‐term illness care,^13^ the effect of workplace conditions on errors,^14^ what they believe may be done to reduce errors,^15^, ^16^, ^17^, ^18^ and how safety problems may impact on their subsequent interactions with the health‐care system.^19^

Although important progress has been made in this area during the last ten years, this is a relatively new field and further research is needed to better understand patients’ perceptions and experiences of safety problems in English general practice. Previous studies are heterogeneous in terms of the different aspects of patient safety examined, but also in terms of countries in which they have been conducted (Australia, New Zealand, USA) with diverse health systems. Patient safety is highly contextual, and findings cannot be necessarily extrapolated across countries. The available evidence in the UK (a country with strong primary care orientation) is still scarce, with only four studies currently published.^12^, ^13^, ^17^, ^20^ Also, previous qualitative studies relied on data obtained through focus groups or individual interviews, including a relatively low number of participants. Additional qualitative research using alternative methodological approaches (eg qualitative content analysis of free‐text responses to a survey completed by a large number of participants) may contribute to a better understanding of patients’ perceptions and experiences of patient safety in primary care.

The aim of this work was twofold: (i) to explore patients’ perceptions and experiences of patient safety in general practices in England and (ii) to identify patient‐centred recommendations to improve patient safety in primary care.

## 2 METHODS

### 2.1 Questionnaire

Data were collected with the Patient Reported Experiences and Outcomes of Safety in Primary Care (PREOS‐PC) questionnaire.^21^ PREOS‐PC was developed in a multistage process supported by an expert panel and informed by two systematic reviews,^22^, ^23^ four focus groups,^17^ 18 cognitive interviews and a pilot study which involved 1975 patients registered in 26 general practices.^21^ Available evidence supports the validity and reliability of the questionnaire.^21^ The standardized items in the survey measure different domains of patient safety including patient and practice activation for patient safety, experiences of safety problems, harm and overall perceptions of patient safety. In addition, PREOS‐PC included seven open questions. Four of them asked patients about their experiences of safety problems and harm, whereas the other three asked about lessons learnt as a result of experiencing a safety problem; good practices followed by health‐care professionals to ensure the provision of safe health care; and suggested changes to improve patient safety in their practices (see Table 1).

**Table 1:** Sociodemographic and clinical characteristics of the respondents and non‐respondents to the seven open‐ended questions included in the PREOS‐PC questionnaire

Open‐ended questions	N	Women (%)	Age (mean (standard deviation))	Educational attainment (% with degree and above)	Health status (% good/ very good)	Long‐term conditions (% one or more long‐term conditions)
1. In case you experienced more than one safety problem in the last 12 months, which of the following better describes the most recent safety problem you experienced? Please select all the boxes that apply to you. Please feel free to describe here in more detail the most recent problem that happened to you.	268	65.5	53.7 (16.9)	41.7	71.7	73.5
2. Please feel free to describe here your experience of being harmed (ie, how your health/wellbeing was affected as a result of a problem with your healthcare).	97	70.8	59.9 (16.1)	46.1	61.5	78.7
3. Were your family/friends affected by the problem? If so, please feel free to describe here how they were affected.	83	70.3	51.8 (15.2)	48.1	58.5	78.5
4. Do you think you have experienced any type of problem or harm as a result of the healthcare provided by your GP surgery before the last 12 months? If so, please describe your experience below (including the approximate date of when the problem happened).	226	58.7	53.5 (15.6)	46.1	73.5	70.7
5. If you have experienced any type of problem or harm as a result of the healthcare provided by your GP surgery either in the last 12 months or before this time, have you learnt anything as a result of that? If so, what have you learnt?	181	61.7	54.5 (16.3)	45.1	74.9	71.9
6. What things, if any, does your practice do well to ensure that care is delivered safely?	452	60.0	56.6 (15.7)	43.8	74.1	74.7
7. What changes, if any, would you suggest to your GP surgery to ensure that care is delivered safely?	422	56.7	55.7 (15.1)	42.9	74.2	72.3
8. Participants not completing any of the seven open‐ended questions	566	57.4	59.6 (16.6)	27.7	74.2	69.5

N, number of respondents.

### 2.2 Data collection

In June 2014, the questionnaire was sent to 6736 adult (18 years old or older) patients from 45 general practices distributed across five regions in the north, centre and south of England. Practices were selected using purposive sampling to ensure variation in terms of list size and levels of deprivation.^21^ Compared to the characteristics of English practices, in general participating practices were larger (mean list size 8744 vs 7041) and had a slightly higher proportion of non‐white ethnicity patients (18.8% vs 15.9%), but were very similar with respect to gender balance (female participants 50.6% vs 49.1%), proportion of older patients (patients aged above 65 16.5% vs 15.3%) and deprivation (Index of Multiple Deprivation^24^ score 25.5 vs 24.0).^25^ Each practice sent the questionnaire with a covering letter and a prepaid return envelope to a computer generated random sample of 150 patients. Ethical approval was granted by Nottingham Research Ethics Committee (Reference 13/EM/0258; July 2013).

### 2.3 Analysis

Firstly, data were cleaned by removing free‐text responses that contained no relevant information eg “N/A” or “No comments.” Clean data were then analysed using conventional content analysis.^26^ A qualitative researcher (LSC) read all data repeatedly to get a clear understanding of the entire dataset^27^ and then coded these data^28^, ^29^ by first highlighting the exact words from the text that appeared to capture key thoughts or concepts. The researcher also made notes of her first impressions and initial analysis. The coding scheme was developed inductively from these data, with codes either coming directly from the text or reflective of one or more key thought(s). An inductive approach was followed because no suitable theoretical framework to test or explore was identified. Key themes were identified in the answers to the open‐ended questions, which were often related. The preliminary coding scheme was discussed with a second researcher (IRC) and revised. All data within each code were re‐examined by the two researchers. Codes then were sorted into categories based on how different codes were related and linked. These emergent categories were used to organize and group codes into meaningful clusters.^30^ All analyses were conducted separately for each question, except for questions 6 and 7 (which were combined because of their substantial overlap in the underlying question). Throughout the analysis process, a third analyst (JMV) was involved for triangulation purposes. A limited number of direct quotes from participants have been used to convey some important themes. Data were analysed using the NVivo 10, a data management and analysis software.

## 3 RESULTS

### 3.1 Response rate

The overall response rate to the PREOS‐PC questionnaire was 18.4% (1244/6736). Compared to the overall characteristics of all eligible patients registered in the 45 participating practices, respondents were more likely to be female (59% vs 51%), aged ≥65 (39% vs 20%) and of white ethnicity (91% vs 82%) (Table 2). A total of 678 patients (55% of those returning completed questionnaires) responded to at least one of the seven open‐ended questions. Those responding tended to be more frequently women, younger, have a worse health status and were more likely to have multiple chronic conditions those not responding to the open‐ended questions.

**Table 2:** Sociodemographic and clinical characteristics of the primary care users who completed the PREOS‐PC questionnaire

	N (%)
Sex^a^	
Male	497 (41.11%)
Female	712 (58.89%)
Age^b^	
18‐34	140 (12.03%)
35‐64	570 (48.97%)
≥65	454 (39.00%)
Ethnicity^c^	
White	1082 (91.15%)
Other ethnic group	105 (8.85%)
Educational level	
Degree, degree equivalent and above	411 (35.16%)
Other qualifications	532 (45.51%)
No qualifications	226 (19.33%)
Health status	
Very good/good	892 (73.54%)
Fair/bad/very bad	321 (26.46%)
Number of long‐term conditions	
0	330 (27.99%)
1	329 (27.91%)
2‐3	366 (31.04%)
>3	154 (13.06%)
Number of medications taken	
0	344 (30.10%)
1‐2	311 (27.21%)
3‐4	222 (19.42%)
>4	266 (23.27%)

a Mean (SD) proportion of female registered in the 45 practices that participated in the study: 0.51 (0.05).

b Mean (SD) proportion of eligible patients aged >65 registered in the 45 practices that participated in the study: 0.20 (0.01).

c Mean (SD) proportion of patients from non‐white ethnicity registered in the 45 practices that participated in the study: 0.18 (0.04).

**Figure 1:**
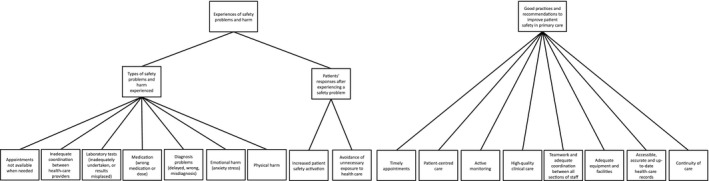
Main themes and subthemes identified

Two main themes were identified (i) experiences of safety problems and harm and (ii) good practices and recommendations to improve patient safety in primary care (Figure 1).

### 3.2 Experiences of safety problems and harm

Two subthemes were identified for experiences of safety problems and harm: types of safety problems and harms experienced, and patients’ responses after experiencing a safety problem.

#### 3.2.1 Types of safety problems and harms experienced

A total of 268 participants responded to at least one of the four open‐ended questions about previous experiences of safety problems or harm. The most frequently reported safety problems (Box 1) were related to the appointment system (n=117), followed by the coordination between health‐care providers (n=46), diagnostic tests (n=41), medication (n=35) and diagnosis (n=32). Harm was generally described in terms of emotional harm (eg anxiety, stress, concern or panic attacks), which were generally produced as a result of delays in obtaining an appointment or in receiving a diagnosis or adequate treatment. Some participants reported that these delays resulted in their condition being unnecessarily extended or exacerbated. In some instances, the harm experienced affected not only the patients but also their families or friends.

Box 1 **Types of patient safety problems experienced**

**Appointment system (N=117)**

Problems with the appointment system included difficulties to get an appointment when needed or when convenient, and difficulties to get an appointment with their preferred GP.

“Availability of appointments is the main problem (…). The on‐line system doesn't work well, neither does booking on the automated telephone system. We are therefore having to ring at exactly 8AM and wait for what seems ages for the phone to be answered and then might be offered an appointment for the following week.” (male, 60 years)

**Coordination between health‐care providers (N=46)**

Patients reported miscommunication and lack of cohesion among staff in the practice and between levels of care. Some participants reported significant delays in being referred to a specialist, which resulted in delayed diagnosis and treatment.

“Following cardiac surgery I was told I should have an annual flu vaccination, staff at the practice have agreed and promised to put me “on the list” several times. Yet I still never get a call and when I enquire I am told there is no note for me to have the vaccination. This has been going on for several years.” (male, 50 years)

**Tests (N=41)**

Problems with tests (eg test inadequately undertaken or results being misplaced) caused delayed diagnosis or treatment. Some patients informed that their tests were done under unsafe conditions, sometimes resulting in avoidable harm.

“I had a swab taken back in January 2014. The doctor said 2 weeks max for results. I phoned no results in. Kept being told they can take up to 2 months. I even came into the practice to chase results. In June they finally admitted my swab was lost.” (female, 27 years)

**Medication (N=35)**

Medication‐related problems included prescribing the wrong type or dose of medication or treatment duration. Patients attributed these problems to clinical or administrative mistakes. They also reported experiences of adverse drug reactions and of GPs expressing reluctance to change their medication plan.

“I was prescribed an old medication which was different to the one I requested. I did not notice until I received my prescription item. This has happened more than once. I have not been believed when I say what I think my problem is. My view was confirmed by a consultant. I changed my GP.” (female, 52 years)

**Diagnosis (N=32)**

Patients reported severe delays in diagnosing their condition. They also reported diagnosis errors (proved by second opinion), which resulted in receiving the wrong treatment.

“I was experiencing severe pain in my upper right leg when sitting and pins and needles in my right foot. I visited on GP who told me not to sit down if it hurt when doing so and she said that she would “sit on it for a while to see if it eased off” It did not so I saw a different GP a couple of weeks later who referred me to a physio‐therapist who then order a scan on my spine where upon it was diagnosed arts having two discs misplaced in my lower spine. I am currently awaiting an appointment with a specialist. I felt that my symptoms were treated flippantly by the first GP I visited I was in obvious pain and had been for several weeks. I felt like she treated my pain as a joke when clearly it was not and was something more serious.” (female 57 years)

**Communication (N=29)**

Lack of patient‐centred communication (eg GPs not listening or believing to their patients, or not explaining to them important aspects concerning their condition, treatment or prognosis) resulted in delayed diagnosis or referrals, which in some instances caused emotional harm to patients.

In addition, confidentiality of information was not ensured by the staff working in the reception area.

“When a new GP joined the surgery, I (or the members of my family) was not informed that they splitting my registered GP's patient list alphabetically and moving me to the new GP's list. The first I realized was when the new GP's details was printed on repeat prescription. On ringing the surgery, I was told that this would make no difference in being able to still see my previously registered GP (for the sake of continuity of being in his case for some years) In reality this has not been the case and I have had a long wait to see the previous GP but could have seen the new GP sooner as he is now the one I am registered with. I remain unhappy about this and feel that the practice manager should have consulted patients before moving them on the list and give them a chance to remain with the GP previously registered with. This may make a difference in an emergency or if I want to see my GP quickly rather than having to book weeks ahead.” (female, 58 years)

**Health records (N=21)**

Health records containing outdated or wrong information caused a delay in diagnosis, prescriptions and reviews. Some patients reported that their health records were not available when needed, which was perceived to be due to fails in transferring the information between levels of care, lack of electronic information and inefficiency in the process of updating records.

“My Drs never has hospital letters available to read as “they take a while to scan in the computer and put on your notes”‐ This is what I'm told even 3 weeks after they have received the letter (I get a copy at the same time) I have to take my copy in and get them to scan it‐ Not sure why!” (female, 33 years)

#### 3.2.2 Patients’ responses to experiencing a safety problem

A total of 181 participants provided information on their responses after experiencing a safety problem in the surgery. Becoming more *active players in their own health care* (increased patient activation)^21^ emerged as the most important response (Box 2). This mainly involved speaking up about concerns they may have had in relation to their symptoms or given diagnosis. Some participants also reported seeking a second opinion when they perceived their problems were not being taken seriously by their GPs, double‐checking the accuracy of the information in their health records or prescriptions and proactively requesting test results or more timely appointments. In addition to this increased proactivity, the *avoidance of unnecessary exposure to health care* emerged as an additional response to a safety problem experience.

Box 2 **Patients’ responses to experiencing a safety problem**

**Increased patient activation levels (N=176)**

Patients who experienced safety problems became more actively involved in ensuring they receive the right health care in the future. This included being more proactive in terms of speaking up about concerns in relation to their health and health care; seeking a second opinion when they felt they needed it; double‐checking the accuracy of written information; requesting test results if delayed; or requesting more timely appointment if necessary.

“Question the doctors and nurses more” (male, 22 years)

“I would persist in asking for further investigations and test to be carried out to try to find out the cause of the health issues” (female, 59 years)

“Make more fuss, be insistent, demand a second opinion” (female, 44 years)

“To insist that I have thorough test if a problem persist so no time is wasted and to trust my instincts and say what I think the problem might be early on even if the GP disagrees” (female, 52 years)

“I now need to check everything that is written about me‐ check reports to hospitals, etc. I can't have the confidence I had in the practice‐ now what I have enjoyed for many years in the past” (female, 71 years)

**Avoid unnecessary exposure to health care (N=6)**

Other patients reported that experiencing a safety problem resulted in them trying to avoid unnecessary exposure to health care.

“Not to have a smear test as I cannot trust the staff to be adequately trained” (female, 62 years)

“Cope on my own” (male, 32 years)

### 3.3 Good practices and recommendations to improve patient safety in primary care

A wide range of factors perceived to mitigate the occurrence of safety problems and harm in general practices emerged from participants’ responses to the questions about “good practices” for safe care (452 respondents) and about “suggestions” to improve safety (422 respondents). Box 3 outlines the main subthemes identified.

Box 3 **Good practices and recommendations to improve patient safety in general practices**

**Access and appointments (N=209)**

“You are able to telephone in if you have an urgent medical problem a nurse will call you back, speak to you, and then will arrange for you to see a doctor that day if she feels the need is urgent, excellent service” (female, 75 years—observed good practice).

“Reduce receptions staffs’ contribution to admin, rather than today's triage status.” (male, 75 years—suggestion to improve safety).

“Improve on‐line and automated systems. Penalise more those who fail to attend appointments” (male, 60 years—suggestions to improve safety).

“Open to longer surgery times plus open on weekends. More doctors needed to reduce waiting times” (female, 59 years—suggestions to improve safety).

**Patient‐centred care(N=66)**

“My GP listens very well to me when I share concerns of my condition. Always is happy to discuss medication + treatment. Allows and helps me to feel very involved in my care and also takes seriously how I fell” (female, 40 years—observed good practice).

“Doctors to listen to patients when speaking‐ not he reading computer screens.” (female, 71 years—suggestion to improve safety).

**Active monitoring (N=64)**

“Regular blood tests, diabetic clinic, practice concerns itself more about my wellbeing than I think I do” (male, 83 years—observed good practice).

“The only thing I could suggest is re: feedback from blood tests etc. it would be good if surgery could ring up and say ‘all clear’ rather than having to assume all is well because you have heard nothing” (female, 62 years—suggestion to improve safety).

“Always follow up important/life changing/emergency appointments” (female, 54 years—suggestion to improve safety).

**Training and technical quality of clinical care (N=50)**

“Evidence based, high standard of protectional care delivered in a friendly setting by a very helpful team of GPs/Nurses and other staff” (male, 54 years—observed good practice).

“Junior doctors [to be] supervised more. I think they have done years of training before they come to our surgery but they still need more supervision” (female, 41 years—suggestion to improve safety).

**Teamwork (N=45)**

“I believe communication between all members of my practice is excellent, leaving me feeling ‘in good hands’ from reception, doctor to pharmacy.” (male, 71 years—observed good practice).

“More conversation and control over the procedures we are sent for in other places.” (female, 48 years—suggestion to improve safety).

**Environment and equipment (N=37)**

“Sanitiser for hands supplied when you walk into the surgery.” (female, 59 years—observed good practice).

“Make sure equipment is working at all times” (female, 43 years—suggestion to improve safety).

**Health records (N=28)**

“I believe the GPs now keep all records on a computer, they can refer to your notes and check they are for the correct person by checking date of birth and address. My GP always explains things thoroughly and is happy to discuss any concerns” (female, 44 years—observed good practice).

“I would suggest that GPs always check the notes of the patient they are seeing and that they listen to what the patient is describing fully” (female, 44 years—suggestion to improve safety).

**Continuity of care (N=19)**

“Provide access to same doctor on return visits” (male, 56 years—suggestion to improve safety).

“To be able to see the same doctor. Then they know your history more than just having a quick glance at medical history” (female, 64 years—suggestion to improve safety).

“Not so many part‐time and short stay doctor‐ very difficult to see your own doctor, only works part time and away on quite a few holidays” (female, 71 years—suggestion to improve safety).

**Seek patients’ feedback (N=4)**

“Perhaps carry out at more frequent surveys with customers such as this one. This is the first I have completed, I think is over 30 years attending the surgery.” (female, 59 years—suggestion to improve safety).

“Make complaints procedure more obvious and accessible.” (female, 45 years—suggestion to improve safety).

*Timely appointments* emerged as an important component of safe health care, being identified both as a good practice and a suggestion for improvement. Participants highlighted the importance of being able to book same‐day appointments when they were ill. They found inefficient those appointment systems operating on a “first ring, first served” basis, which required them to contact their practice early in the morning to get an appointment and to wait on the telephone line often enduring lengthy delays. Participants’ suggestions for addressing this issue included extra telephone lines, increased staffing numbers and automated or online appointment systems. Availability of prompt emergency appointments (especially for children and maternity care) and access to nursing care were regarded as important components of safe care. Participants proposed longer opening hours and weekend appointments. They proposed telephone consultations (reported as a good practice by some participants) as a way of getting timely access to their providers.

Participants perceived *patient‐centred communication* as a key aspect for safer health care. This was perceived as listening attentively to the patient, allowing enough consultation time for effective communication, creating an environment in which patients feel comfortable speaking up about their concerns, and ensuring all the relevant information (including treatment options and potential drug side‐effects) is provided to the patients. It was important for some participants to feel “listened to” by their GP because it made them feel more confident with the care they received. Some participants suggested staff changes (employing more qualified staff) or increased training (especially for receptionists and junior staff) to improve their empathy, confidentiality and respectfulness.

Participants perceived that the provision of *high*‐*quality clinical care* was important to prevent safety problems and harm. Efficient, knowledgeable professional staff who kept up to date with policies and protocols and who provided evidence‐based health care were perceived to be better equipped to provide safer health care. Participants linked efficiency with *active monitoring,* which was conceptualized in terms sending regular reminders to patients for tests and follow‐ups, calling with test results, and following up medication, test results and patients’ progression of their condition.

*Teamwork,* cohesion, good relationships, coordination and communication between all sections of staff within the practice were perceived as important contributors of safe health care. Staff working together as part of a *coordinated team* was linked to safe care especially when different professionals were involved in the care. Better communication and control over procedures were common recommendations.

The practice *environment* was another factor considered to influence patient safety. This included having clean, open and pleasant areas with accessible facilities (eg, an automatic door entry or information displayed in screens in the waiting room). Staff hand washing and the storage of medications in safe places were also perceived as important elements of patient safety. Some participants suggested changes in practices’ *material resources and facilities*, such as a bigger car park, cleaner sign posting, more equipment and variety of tests and medication availability.

Easy and quick access to accurate and up‐to‐date *health‐care records* emerged as good practice to ensure safe care. Some patients reported how their surgeries had a process of keeping their records up to date by regularly double‐checking their personal details (ie name and date of birth of the patient) with them, as well as their clinical information. Participants also mentioned the importance of pharmacies in double‐checking the accuracy of their health‐care records when dispensing medication, and underscored the positive role of IT system in this issue.

*Continuity of care* emerged as an important factor related to patient safety. Participants perceived their usual doctors to be more familiar with their own medical history and therefore less prone to diagnosis or treatment‐related errors. They suggested that in those cases where continuity of care cannot be offered, GPs should carefully review patients’ health records prior to the consultation.

Some participants suggested that a structured *engagement* and feedback from patients should be encouraged to achieve safer health care.

## 4 DISCUSSION

This qualitative study examined patients’ perceptions and experiences of safety problems in general practices in England and identified a number of different factors that were perceived to affect safety as well as the lessons learnt by patients after experiencing safety problems. They provide a comprehensive and sophisticated set of patient‐based recommendations on how to improve patient safety for patients (increasing patient activation and reducing unnecessary care) and for GP practices in relation to consultations (focussing on communication, ensuring continuity and proactive monitoring), and the general organization (appointments, health records, teamwork and technical professional competence) and environment of the practice.

### 4.1 Strengths and limitations

In contrast with previous qualitative studies on primary care patient safety, this study is based on data from a large number of participants from 45 practices distributed across England. The study also builds on and expands previous related research focusing on patients’ perceptions of safety in primary care^12^, ^13^, ^16^, ^17^, ^20^, ^22^, ^31^, ^32^ that has resulted in the development of a patient (rather than a professional) model of patient safety. Some limitations need to be acknowledged as well, mainly in terms of representativeness. Although each practice selected a random sample of patients, the response rate to the questionnaire was low with some patient groups being underrepresented, especially male and younger patients. Moreover, 46% of the people completing the questionnaire did not answer any of the open‐ended questions and those who did had higher educational attainment. This may have introduced a bias (selection bias) if the experiences and perceptions of patients with higher educational attainment differ from those from patients with lower educational attainment.

### 4.2 Comparison with previous literature

The type and nature of safety problems and harm experienced by patients are generally in line with previous qualitative work^13^, ^16^, ^17^, ^32^ and with the quantitative data from the PREOS‐PC survey.^25^ Problems related to access to health care were very frequently reported. This may be partially explained by the fact that this study was conducted during a period of economic austerity in England, and the financial cuts imposed in health‐care provision could have possibly affected more severely access than other areas of safety. This hypothesis is supported by data from the GP Patient Survey (a survey measuring patient experiences in family practices in England, mailed each year to 2.7 million patients),^33^ which shows an increase in the percentage of patients who had to wait more than 1 week for an appointment over the last four years (from 13% in 2012 to 18% in 2015).^34^

The most important lesson patients reported to have learnt as a result of experiencing a safety problem was to increase their proactivity to prevent potential safety problems happening in the future. Patients reported changing their role as recipients of health care, and instead proactively spoke up, sought a second opinion or requested timely test results. There is a growing interest in increasing patient activation to achieve safer health care,^10^, ^11^, ^35^, ^36^, ^37^, ^38^, ^39^ and our findings support the idea that higher patient involvement is feasible, acceptable by patients, and perceived by them as an important strategy for harm prevention. Some patients who experienced safety problems changed their health‐care‐seeking behaviour toward the avoidance of unnecessary exposure to health care. In general, our results resonate with a previous study which observed four different types of responses to safety problems in primary care: avoidance (eg stop going to the doctor); accommodation (eg learn to deal with delays); anticipation (eg attend to details, acquire knowledge, and actively communicate); and advocacy (eg get a second opinion).^19^ Our findings provide the basis for the development of a framework on recommendations for patients about how they could contribute to improve patient safety (Box 4).

Box 4 **Framework on recommendations for patients on how to contribute to patient safety**

1

- Double‐check the accuracy of written information and given medication.
- Be proactive in:
  ∘ speaking up about concerns in relation to your health and health care;
  ∘ requesting test results if delayed;
  ∘ requesting longer appointment if necessary.
- Seek a second opinion if you feel you need it.
- Reduce unnecessary appointments.

We deliberately asked participants both about good practices for safer health care and suggestions for improving safety as this enabled us to obtain a better understanding of the factors that patients felt contributed to, or mitigated against, the occurrence of safety problems and harm in their practices. Timely access to primary care consultations emerged as the most important factor, both in terms of good practices and suggested changes. A substantial number of patients expressed concerns about the system to book appointments. Most of these events have been traditionally regarded as service quality incidents. However, in the primary care context, access is considered an important determinant of safety.^13^, ^14^, ^20^

The provision of high‐quality clinical care was perceived by patients to have a strong influence on patient safety. In most cases, it was clearly distinguished by patients from patient‐centred care. In addition, a frequent suggestion to improve safety was to increase the training of practice staff. These findings suggest that patients were (i) aware of the importance of high‐quality clinical care to prevent safety incidents and (ii) able to make their own judgements on the clinical quality of the health care they received. Our results resonate with a recent systematic review examining patients’ views of adverse events in primary care, which suggested that patients are able to identify events traditionally recognized as technical medical aspects such as errors in diagnosis.^32^ The criteria used by patients to make such judgements remain unclear, so does the extent to which they can be used to predict health outcomes. These aspects represent areas for future research.

Patient‐centredness was also frequently perceived by patients as an important determinant of safety, being a recurrent subtheme both as a suggestion to improve safety and as good practice. The importance of patient‐centred communication to prevent errors and harm has been previously highlighted both by patients^13^, ^17^, ^31^, ^40^ and by clinicians.^31^, ^41^, ^42^ Effective communication could have a number of positive consequences, for example, preventing the likelihood of adverse events, reducing psychological distress for the patients, increasing patient satisfaction, misinterpreting or reducing the likelihood of incorrect diagnosis or treatment,^43^ and possibly decreasing the potential number of malpractice claims.^32^

Active monitoring emerged as an additional feature of patient safety. Patients felt anxious when they could not get access to their test results because they felt at risk of receiving a delayed diagnosis and treatment. Research has shown that patients may lack an understanding or awareness of the results handling process in their practice and that they usually do not contact their practice for test results, unless they considered themselves to be ill.^44^

Continuity of care also emerged as an important attribute of patient safety in primary care, which supports results from previous research.^17^, ^20^ Ensuring that patients are able to see the same GP enables the GP to become a repository of information; acquire specialist knowledge of a patient's condition; become familiar with the patient's consulting behaviour; provide holistic care; and foster the development of trust.^45^

### 4.3 Practice implications

Health‐care professionals and commissioners of English general practices should be aware of the factors that patients perceive can influence safer health care. Even though patients’ perceptions of safety problems may not always result in adverse events, they could, however, influence patient satisfaction, which has been associated with a higher engagement of health services and increased treatment adherence.^46^, ^47^ Practices should therefore consider implementing evidence‐based strategies to improve patient perceived safety.^11^ Although the evidence base of interventions to improve safety in general practices is still scarce, most of the factors identified by patients were specific and fall within the realm of health‐care quality, where available evidence is stronger.^2^ For example, evidence suggests that GP or nurse‐led telephone triage could be effective to improve access to same‐day consultations,^48^ which is one of the most frequent recommendation from patients to achieve safer health‐care delivery.

Patients made a large number of recommendations to improve different areas of patient safety in general practices. Research is now needed to explore the acceptability and perceived utility of those recommendations by health‐care professionals and commissioners; to identify effective strategies to support their implementation in a context of resource limited service; and to measure its impact.

Finally, practices may be heterogeneous in terms of the areas they need to improve to deliver safer health care (eg, some practices may be perceived to provide excellent patient‐centred care, but struggle to offer timely appointments, or vice versa). The use of standardized and validated patient reported instruments, such as the Patient Reported Experiences and Outcomes of Safety in Primary Care (PREOS‐PC) questionnaire,^10^ might prove a valuable resource for practices in order to help them identify and prioritize areas for safety improvement.

## 5 CONCLUSION

This study identified a number of key areas that patients believed influenced the safety of health care provided in their general practices—namely access to appointments, quality of clinical care, psychosocial relationship with health‐care providers or continuity of care—and helped us increase our understanding of patients’ behavioural responses to experiences of safety problems and harm. The information gathered in the open‐ended questions complemented the quantitative data generated from the standardized items in the questionnaire,^25^ allowing us to better understand specific aspects of patients’ experiences and perceptions of safety problems in general practices in England.

## CONFLICT OF INTEREST

No conflicts of interest have been declared.
